# A Novel Risk Model Identified Based on Pyroptosis-Related lncRNA Predicts Overall Survival and Associates With the Immune Landscape of GC Patients

**DOI:** 10.3389/fgene.2022.843538

**Published:** 2022-02-07

**Authors:** Tingting Xu, Hanxin Gu, Changsong Zhang, Wushuang Zhang, Xiaolong Liang, Xiaoxia Cheng

**Affiliations:** ^1^ The Affiliated Suzhou Science and Technology Town Hospital of Nanjing Medical University, Suzhou, China; ^2^ Department of Gastrointestinal Surgery, the First Affiliated Hospital of Chongqing Medical University, Chongqing, China

**Keywords:** gastric cancer, pyroptosis, prognosis, immune, tumor mutation burden

## Abstract

Gastric cancer (GC) is one of the most common malignant gastrointestinal tumors worldwide. Pyroptosis was widely reported to exert a crucial function in tumor development. In addition, pyroptosis was also proved to be associated with the immune landscape. However, whether pyroptosis-related lncRNAs are associated with the prognosis and the immune landscape of GC remains unclear. In the present study, we first constructed a novel risk model by using pyroptosis-related lncRNAs. We identified 11 pyroptosis-related lncRNAs for the establishment of the risk model. The risk model could be used to predict the survival outcome and immune landscape of GC patients. The results of survival analysis and AUC value of a time-related ROC curve proved that our risk model has an elevated efficiency and accuracy in predicting the survival outcome of patients. We also found that the risk model was also associated with the immune landscape, drug sensitivity, and tumor mutation burden of GC patients. In conclusion, our risk model plays a crucial role in the tumor immune microenvironment and could be used to predict survival outcomes of GC patients.

## Background

Gastric cancer (GC) remains to be one of the most lethal malignant tumors, with an estimated over one million new cases and 0.76 million new deaths worldwide annually (Sung et al., 2021). Despite the improvement of diagnosis and treatment in GC, the overall survival of GC patients, especially for advanced gastric cancer (AGC) patients remains poor (Smyth et al., 2020). The traditional classification of gastric cancer is based on the histological classification systems, which is controversial in predicting the prognosis of patients (Smyth et al., 2020). Recently, the Cancer Genome Atlas (TCGA) proposed a new classification strategy based on the DNA copy number alterations, mutations, mRNA, miRNA, and protein expression patterns of GC patients and identified four subtypes GC: genome stability, microsatellite instability (MSI), chromosomal instability (CIN), and EBV positive (EBV) (The Cancer Genome Atlas Research Network, 2014). The molecular analyses regarding GC could help to identify novel prognostic biomarkers and might guide future treatment development.

Long noncoding RNAs are a class of non-protein-coding transcripts with a length longer than 200 nucleotides (Sun et al., 2018). Substantial evidence suggests lncRNAs exert vital roles in diverse biological processes (Guttman et al., 2011; Ulitsky et al., 2011; Sauvageau et al., 2013). Recent studies revealed that various lncRNAs also exert crucial functions in numerous biological processes concerned with tumorigenesis (Peng. W.-X et al., 2017; Chi et al., 2019). Furthermore, lncRNAs were widely reported to modulate pyroptosis in many diseases including cancer (Chen et al., 2021; Huang et al., 2021; Ning et al., 2021; Wang. L.-Q et al., 2021). For example, lncRNA HOTTIP was reported to inhibit the pyroptosis of ovarian cancer cells via targeting miR-148a-3p/AKT2 axis (Tan et al., 2021). Knocking down of LncRNA-XIST could inhibit pyroptosis by affecting miR-335/SOD2/ROS signal pathway in non-small cell lung cancer (NSCLC) (Liu et al., 2019). LncRNA GAS5 exerted a tumor-inhibiting function in ovarian cancer, which was reported to be associated with pyroptosis (Li et al., 2018). LncRNA XLOC_000647 was reported to inhibit the progression of pancreatic cancer through down-regulating pyroptosis-related gene NLRP3 expression (Hu. H et al., 2018). These findings indicated that lncRNAs were closely correlated with pyroptosis.

Pyroptosis was originally condemned as apoptosis because its characteristics are similar to apoptosis. Subsequently, pyroptosis was identified to be pro-inflammatory programmed cell death, which makes it distinct from apoptosis (D'Souza and Heitman, 2001). Pyroptosis was reported to exert a critical role in multiple cancers including gastric cancer. It has been found that pyroptosis-related genes such as NALP1, GSDMD, and GSDMB were abnormally expressed between normal tissues and tumor tissues, and these genes could regulate the biological function of cancer cells (Komiyama et al., 2010; Chen et al., 2015; Gao. J et al., 2018). Pyroptosis-related genes were considered as potential therapeutic targets for multiple tumors (Chen et al., 2015; Miguchi et al., 2016; So et al., 2018). In gastric cancer, pyroptosis-related genes such as GSDMA, GSDMB, and GSDMC were revealed to be abnormally expressed in GC and associated with tumor progression (Saeki et al., 2009; Komiyama et al., 2010; Qiu et al., 2017). Pyroptosis-related genes could also be used as novel prognostic biomarkers for tumors (Lin et al., 2021; Ye et al., 2021). More recently, pyroptosis was uncovered to be associated with the immune landscape in tumors. Pyroptosis could cause antitumor immunity both in primary and metastatic tumors (Zhao et al., 2020). It was reported that pyroptosis can induce inflammation and produce antitumor immunity, which could work in synergy with checkpoints blockade (Wang et al., 2020). In addition, the tumor microenvironment, especially for the infiltration of CD4^+^ and CD8^+^ T cells, could be regulated by the mutant MEK and BRAF inhibitors via pyroptosis (Erkes et al., 2020). These findings indicated that pyroptosis plays a vital role in tumors, especially for the tumor immune microenvironment. However, the relationship between pyroptosis-related lncRNAs and the immune landscape in GC remains largely unknown.

In the present study, we identified 11 pyroptosis-related lncRNAs for the establishment of the risk model. We explored the prognostic function of the risk model in patients with GC. We also determined the correlation between risk model and immune cell infiltration, expression of immune checkpoint genes, immunotherapy score, and tumor mutation burden in GC patients. As expected, we revealed that our risk model exhibits a preferable performance in predicting the survival outcome of GC patients. Our risk model showed an advanced efficiency in predicting the immune cells infiltration, immunotherapy effectiveness, and tumor mutation burden of GC patients.

## Results

### Establishment of the Prognostic Signature

**FIGURE 1:**
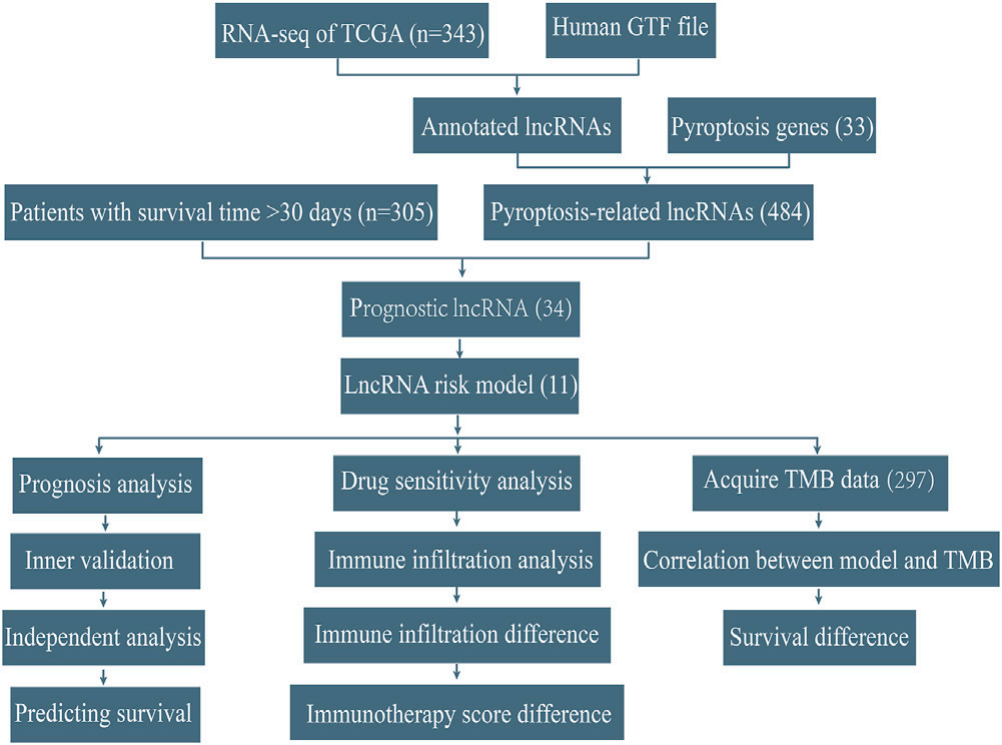
Workflow of this study.

**FIGURE 2:**
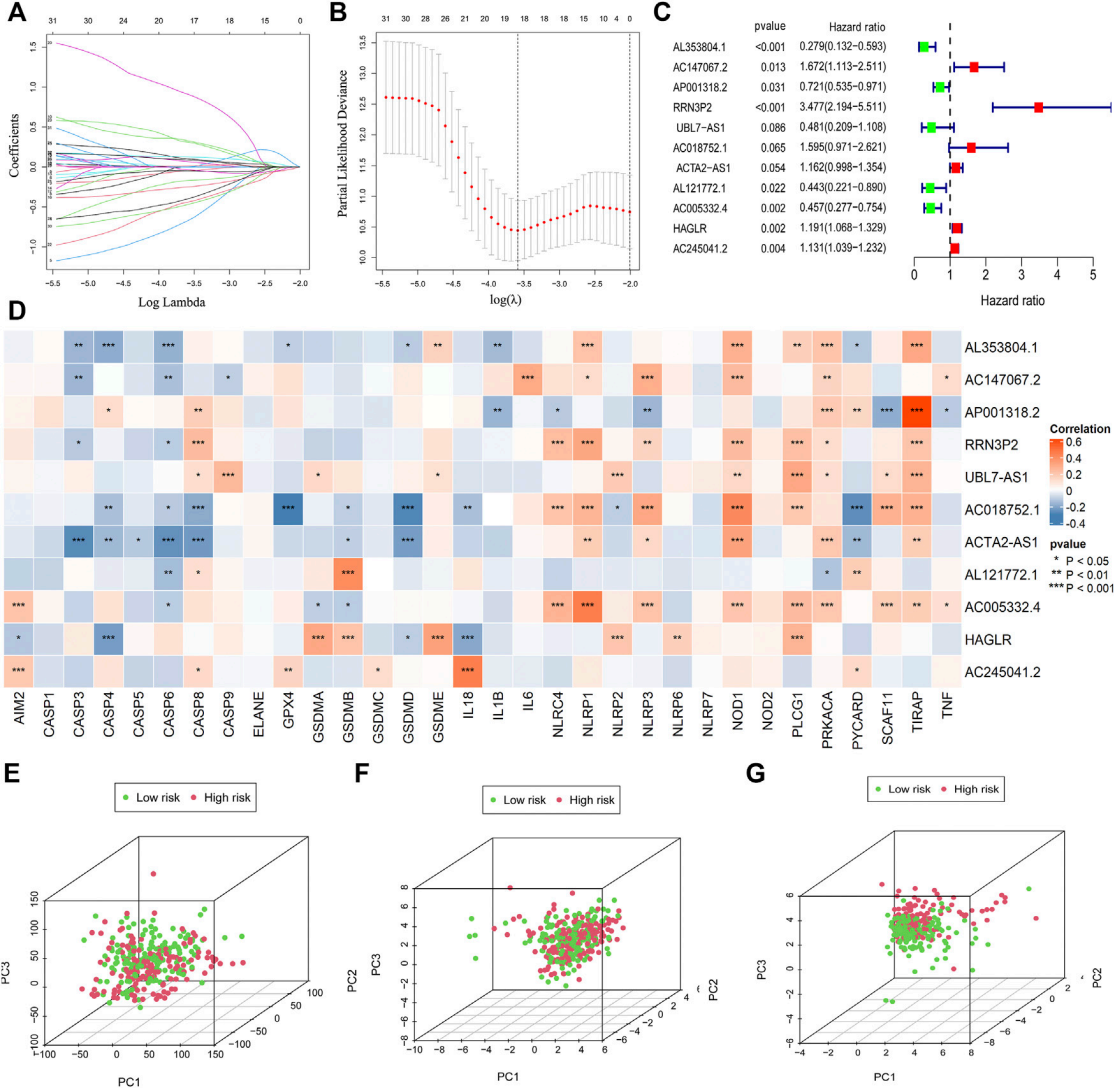
Establishment of the risk model. The LASSO and multi-cox analysis were performed to construct the risk model **(A–C)**. 11 pyroptosis-related lncRNAs were screened for the construction of the risk model **(C)**. The correlation between 33 pyroptosis genes and 11 lncRNAs in the risk model was visualized **(D)**. PCA analysis was conducted for the entire gene set **(E)**, 33 pyroptosis genes **(F)**, and 11 pyroptosis-related lncRNAs in the risk model **(G)**.

The workflow was shown in Figure 1. We acquired the expression matrix and relevant clinical data of 343 gastric cancer patients from the TCGA database. By using the human GTF file, we annotated the gene symbols and obtained the expression matrix of mRNA and lncRNA. We extracted the expression data of 33 certified pyroptosis genes from the mRNA matrix. Then, co-expression analysis was conducted between 33 pyroptosis genes and lncRNAs to acquire pyroptosis-related lncRNAs. We obtained 484 pyroptosis-related lncRNAs for subsequent analyses. To identify prognostic pyroptosis-related lncRNAs, we combined the survival information of GC patients with lncRNA expression data. Then, we randomly divided 305 GC patients into two sets (training set and testing set). Univariate analysis was performed on the training set, which result in obtaining 34 prognostic pyroptosis-related lncRNAs (Supplementary Table S1). The result of the univariate analysis is shown in Supplementary Table S2. To reduce the number of genes, we further conducted lasso regression analysis and obtained 17 prognostic pyroptosis-related lncRNAs (Figures 2A,B, Supplementary Table S3). The coefficient value of 17 lncRNAs result from lasso regression analysis was exhibited in Supplementary Table S4. Subsequently, the multi-cox analysis was conducted on 17 prognostic pyroptosis-related lncRNAs. The risk score value was calculated according to the following formula (n, k, *coef value,* and *expression* represent for the number of lncRNAs, selected lncRNA, regression coefficient value, and lncRNA expression value, respectively.):

$$Risk\;score=\sum_{k=1}^{n}coef\;value\left(gene\;k\right)*expression\left(gene\;k\right)$$

The median value of the risk score is 1.0076. A total of 11 pyroptosis-related lncRNAs were screened for the construction of the risk model (Figure 2C; Supplementary Table S5).

The correlation between 33 pyroptosis genes and the 11 lncRNAs was evaluated and visualized (Figure 2D).

In order to verify whether the risk model could distinguish the low-risk and high-risk patients in the entire set, we conducted principal component analysis according to the risk patterns of the patients. Results demonstrated that only 11 lncRNAs in the risk model showed elevated efficiency in separating patients with different risk patterns (Figures 2E–G).

### Survival Analysis Based on the Model

**FIGURE 3:**
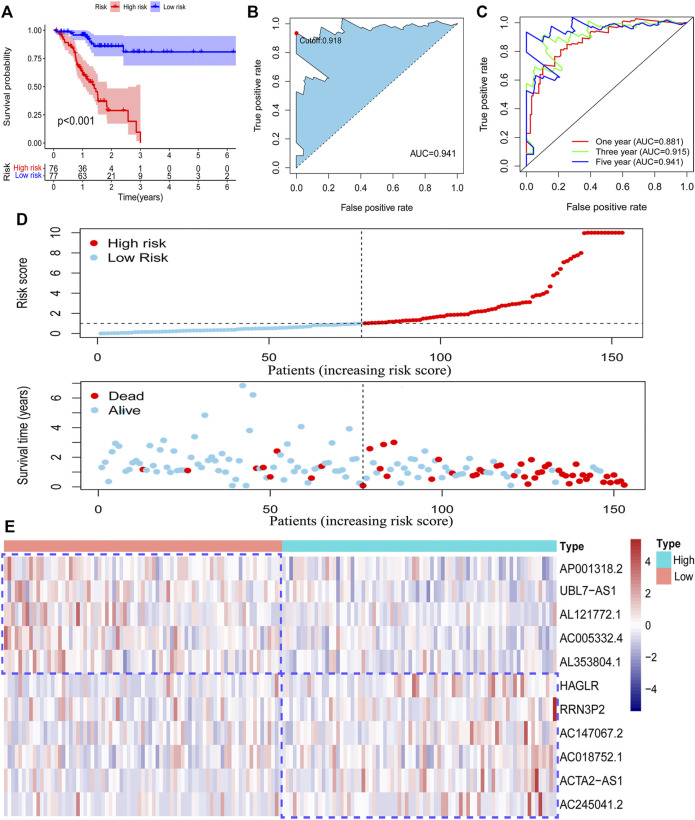
Survival analysis of the risk model. The survival difference between the low-risk patients and high-risk patients in the training set **(A)**. A time-dependent ROC curve was plotted to test the accuracy of the risk model **(B, C)**. Patients in the training set were ranked according to the risk score. Then, the survival status difference of the patients between the two groups was visualized **(D)**. The RNA level of the 11 pyroptosis-related lncRNAs in the training set was visualized **(E)**.

To validate the function of the risk model in predicting the survival outcome of GC patients in the training set, we performed survival analysis and observed that patients in the high-risk group have poorer survival outcomes than those in the low-risk group (Figure 3A). We also evaluated the accuracy of the risk model by using a ROC curve. The minimum AUC value exceeded 0.85, which confirmed that the risk model was efficient in predicting patient survival outcomes (Figures 3B,C). We also found that patients in the high-risk group had higher mortality than those in the low-risk group (Figure 3D). The RNA level of 11 pyroptosis-related lncRNAs in the risk model was visualized (Figure 3E). These findings suggested that our risk model has a preferable performance in predicting the survival of GC patients.

### Inner Validation of Risk Model

**FIGURE 4:**
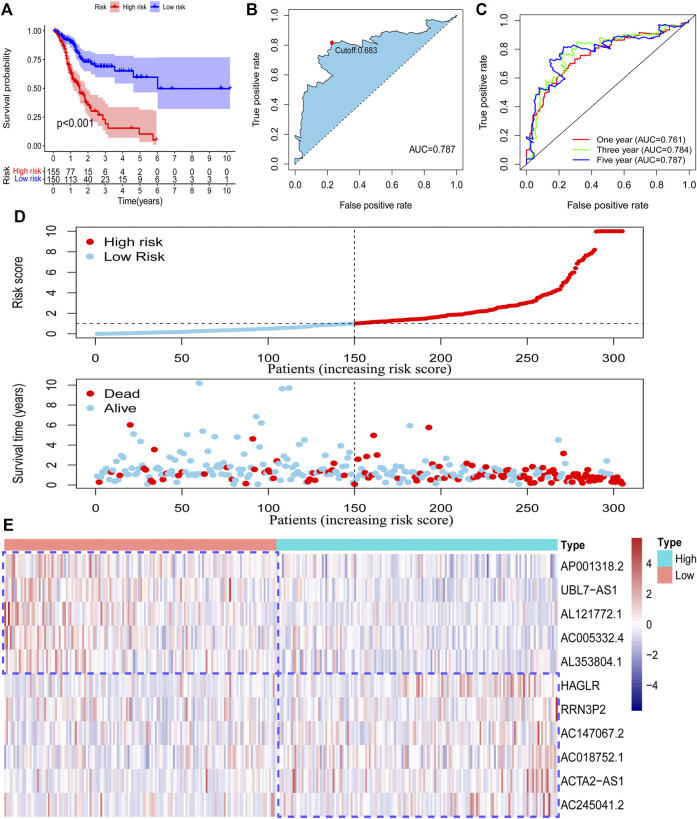
Validation of the risk model in the entire set. The survival difference between the low-risk patients and high-risk patients in the entire set **(A)**. A time-dependent ROC curve was plotted to test the accuracy of the risk model **(B, C)**. The survival status difference of the patients between the low-risk and high-risk groups was visualized **(D)**. The RNA level of the 11 pyroptosis-related lncRNAs in the entire set was visualized **(E)**.

To confirm our risk model could be used to predict the prognosis of all acquired patients, we further conducted survival analysis on patients enrolled in the testing set and entire set. As expected, we found that all low-risk patients in the two sets had superior survival outcomes to the patients under high-risk (Figure 4A; Supplementary Figure S1A). The AUC value of the ROC curve also suggested that the risk model has a preferable efficiency in two validation sets (Figures 4B,C; Supplementary Figures S1B,C). We also observed that there were more deaths in high-risk patients than in low-risk patients in two validation sets (Figure 4D; Supplementary Figure S1D). The RNA level of 11 pyroptosis-related lncRNAs in two validation sets was visualized by using a heatmap (Figure 4E; Supplementary Figure S1E). These findings suggested that the risk model could also be used to predict the survival outcomes of GC patients in the testing set and the entire set.

### Prognostic Value of the Risk Model

**FIGURE 5:**
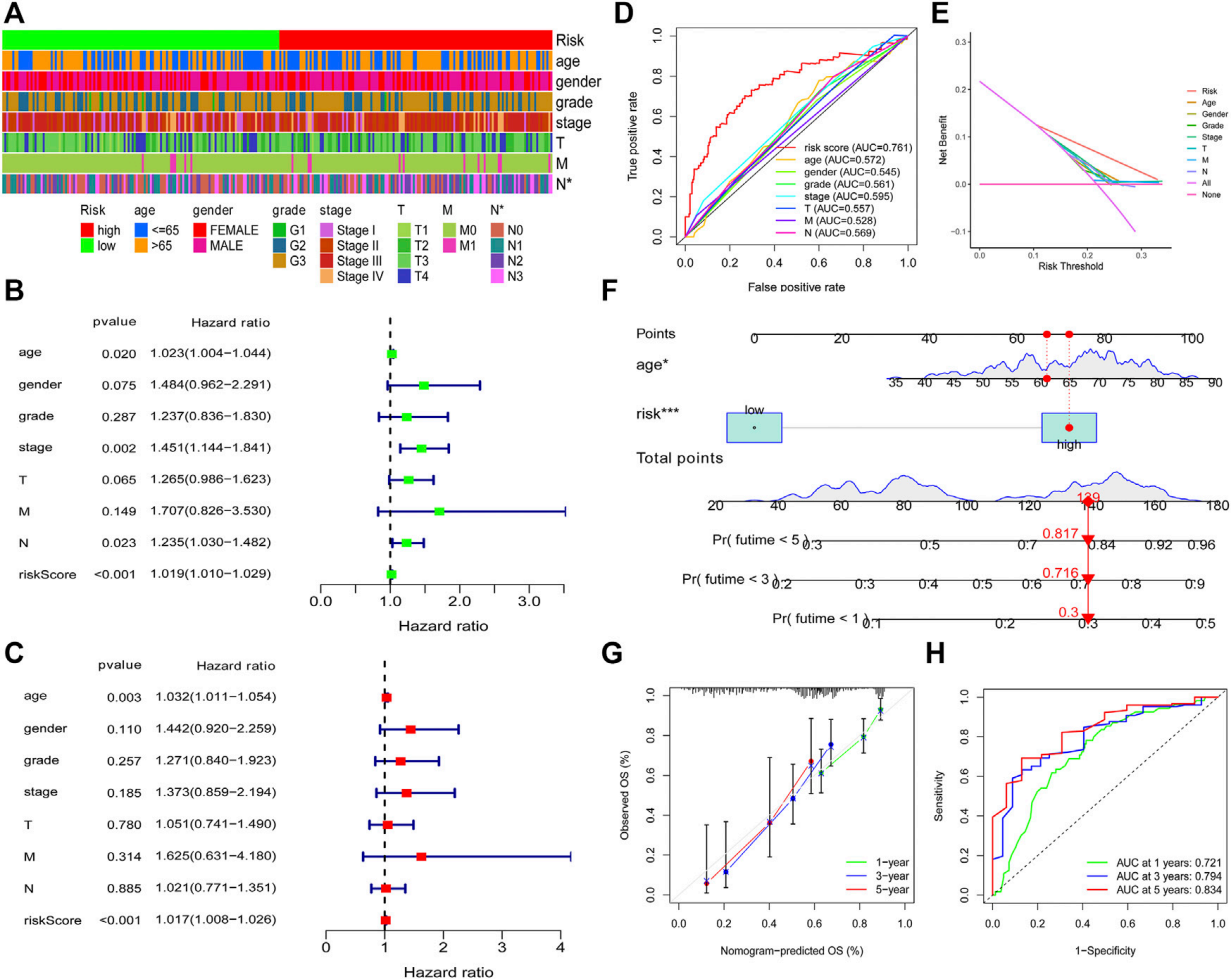
Clinical application of the risk model. Correlation between the risk model and the clinicopathological characteristics of GC patients **(A)**. Univariate analysis and multivariable analysis were conducted to verify the independent prognosis function of the model **(B, C)**. ROC and DCA curves were performed to confirm the superiority of the risk score in clinical application **(D, E)**. The nomogram was plotted to obtain the predicting survival time of GC patients **(F)**. A calibration curve was utilized to assess the accuracy of the model in predicting patients’ survival time **(G)**. The AUC value of the nomogram **(H)**.

To explore the relationship between the model and clinical characteristics of GC patients, we conducted a chi-square analysis and found that low-risk patients had a lower N stage than high-risk patients (Figure 5A), which suggested that low-risk patients might have lower lymph node metastasis. Then, univariate analysis and multivariable analysis were conducted to determine the independent prognostic function of the model. Results demonstrated that the risk score could be used as an independent prognostic indicator (Figures 5B,C). To further confirm that the risk score is superior to other clinical characteristics in predicting patient survival outcomes, a clinically relevant ROC curve and a decision curve were plotted. We observed that risk score had a superior efficiency in predicting patient survival outcomes (Figures 5D,E). In addition, a nomogram was plotted to obtain the predicted survival time of GC patients (Figure 5F). We also constructed a calibration curve and a ROC curve to determine the accuracy of our nomogram at 1, 3, and 5 years, respectively. Results demonstrated that the actual survival time of patients was almost consistent with the predicted survival time (Figures 5G,H).

**FIGURE 6:**
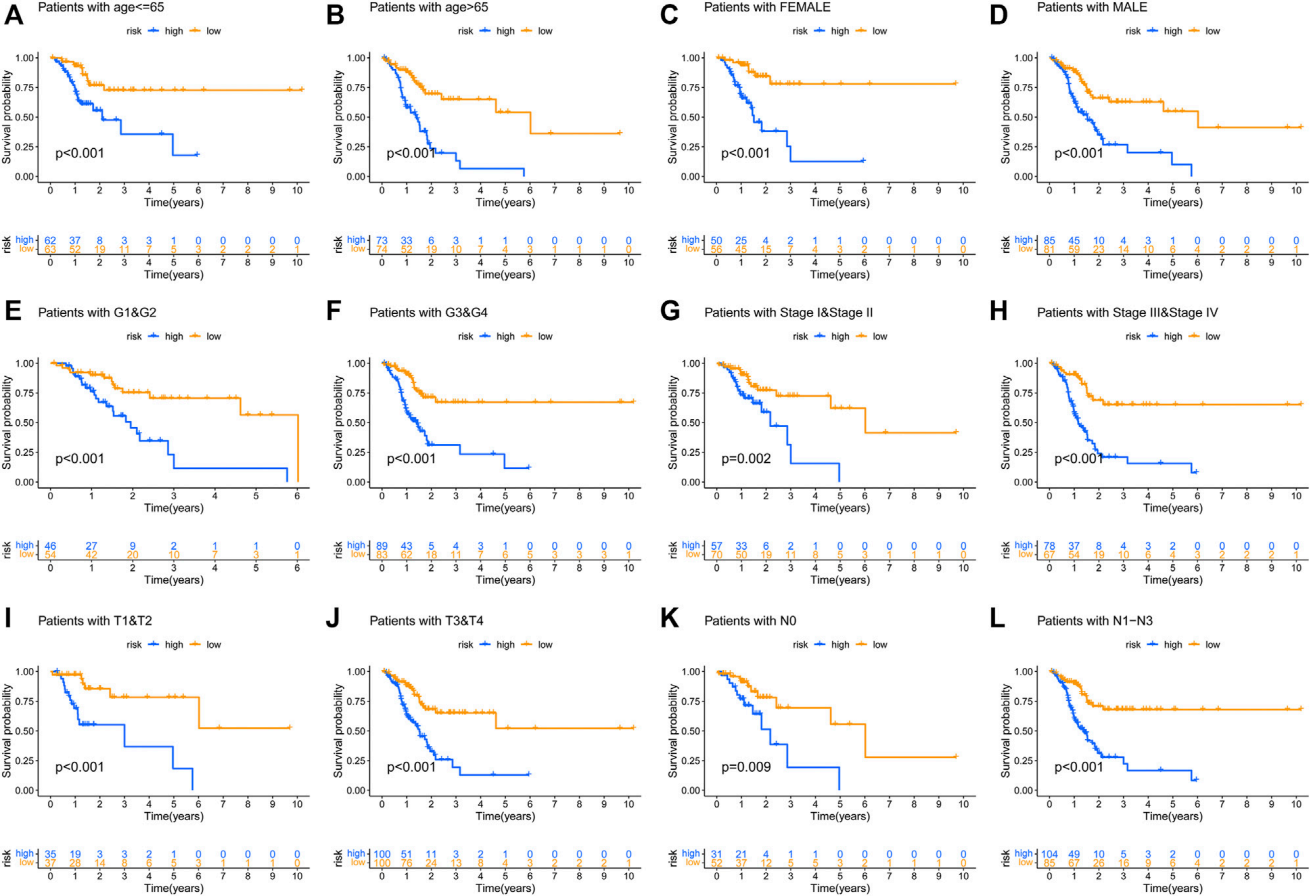
The prognostic function of the risk model in patients with various clinicopathological characteristics was assessed **(A–L)**.

To prove the prognostic effectiveness of the risk model in patients with diverse clinical characteristics, we divided all patients into two subgroups according to different clinical characteristics and evaluated the survival differences of GC patients. Interestingly, we observed that low-risk patients in all subgroups had better survival outcomes than high-risk patients (Figures 6A–L).

### Relationship Between the Model and Immune Signature

**FIGURE 7:**
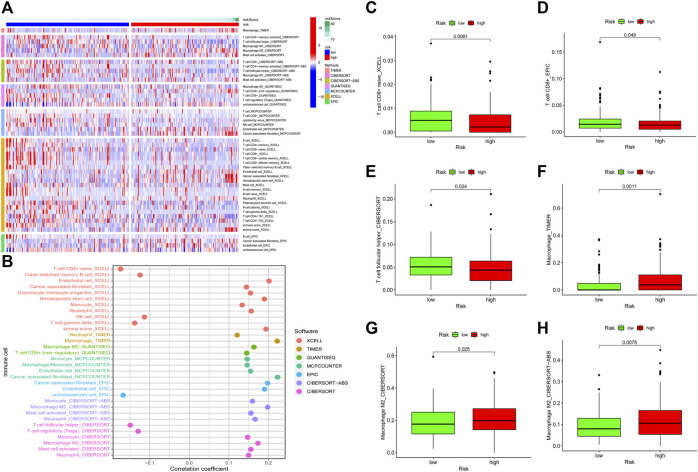
Relationship between the risk model and tumor immune infiltration. The infiltration status of immune cells between the low-risk patients and the high-risk patients **(A, B)**. Differences in the infiltration of T cell CD8^+^ cells, T cell follicular helper, macrophage, and M2 macrophage between the low-risk group patients and the high-risk group patients were visualized **(C–H)**.

To detect the relationship between the risk model and tumor immune microenvironment, we download the TCGA tumor immune infiltration data from TIMER2.0. Subsequently, we visualized the differences of immune infiltration cells between the low-risk patients and the high-risk patients by using a heatmap and a bubble graph (Figures 7A,B). We observed that there was more infiltration of NK cell and T cell in low-risk patients (Figure 7B). However, high-risk patients have a higher infiltration of macrophages, monocyte, mast cells activated, and neutrophils (Figure 7B). We visualized the differences in the infiltration of T cell follicular helper, T cell CD8^+^, M2 macrophage, and macrophage between two groups (Figures 7C–H). These results indicated that the risk model could be used to predict the immune signature of GC patients.

### Clinical Application of the Risk Model

**FIGURE 8:**
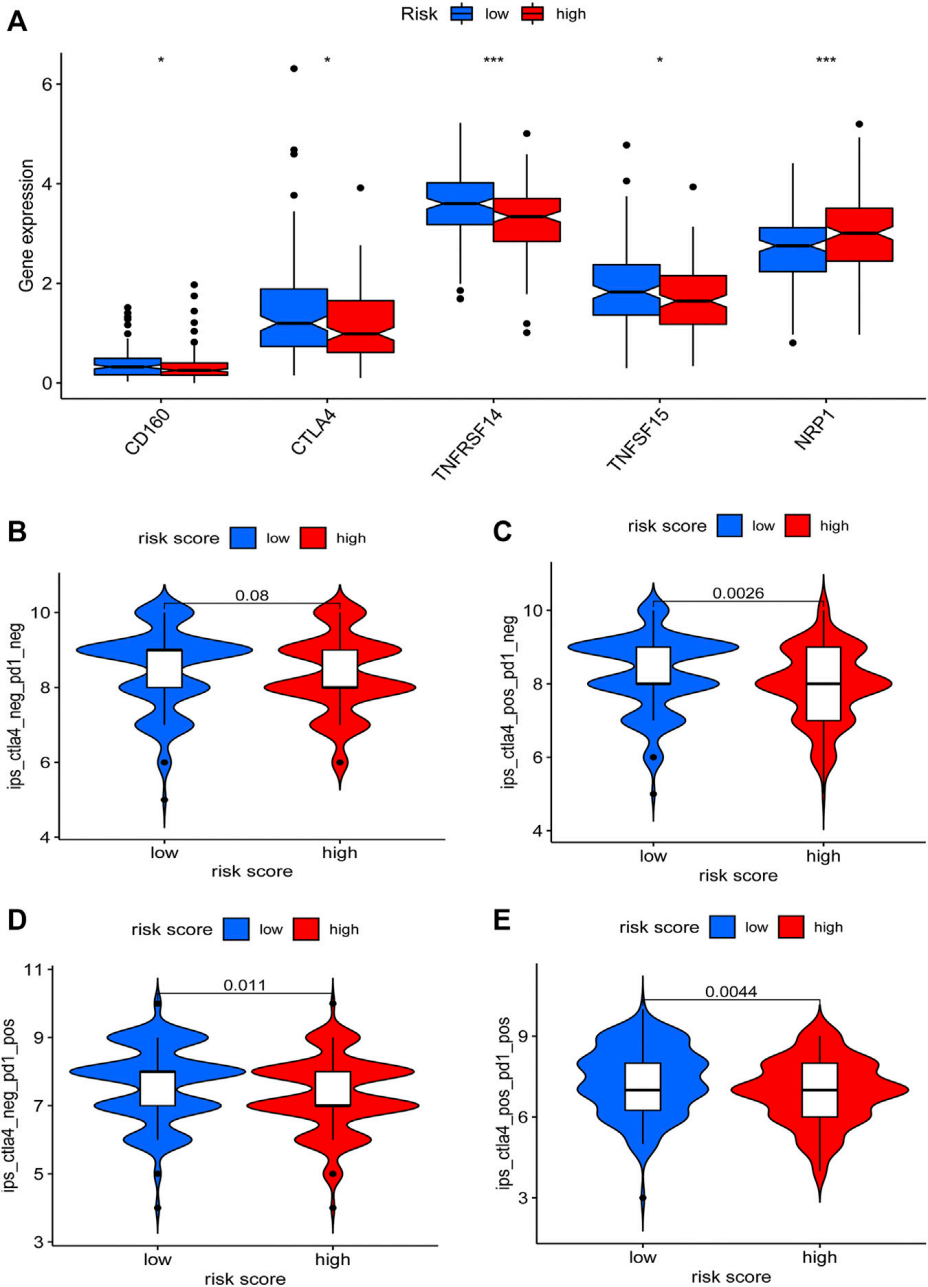
Relationship between the risk model and immunotherapy. Expression differences of immune checkpoint genes between the low-risk and the high-risk patients were determined **(A)**. The immunotherapy scores difference of GC patients with different status of CTLA4 or PD-1 between two groups were visualized **(B–E)**.

To explore the clinical application value of the model, we determined the sensibility differences of 138 drug chemotherapies/targeted therapies between low-risk and high-risk patients. Results demonstrated that patients with a higher risk score might be more sensitive to imatinib, dasatinib, etc., while patients with a lower risk score might be more sensitive to ABT.888 (PARP inhibitor) (Supplementary Figures S2A–L). In terms of immunotherapy, we compared the expression level of immune checkpoint genes between low-risk and high-risk patients. We found that the mRNA level of CD160, CTLA4, TNFRSF14, and TNFSF15 were higher in the low-risk patients (Figure 8A). Furthermore, we download the immunotherapy score data from TCIA (https://tcia.at/) database and compared the difference in immunotherapy scores between the two groups. Interestingly, we observed that there was no difference in immunotherapy scores between CTLA4 and PD-1 double-negative patients. However, low-risk group patients with single positive of CTLA4 or PD-1 and double-positive of CTLA4 and PD-1 had higher immunotherapy scores (Figures 8B–E). These findings proved that our risk model could also be used to predict the therapeutic benefits of GC patients.

### Correlation Between the Risk Model and Tumor Mutation Burden

**FIGURE 9:**
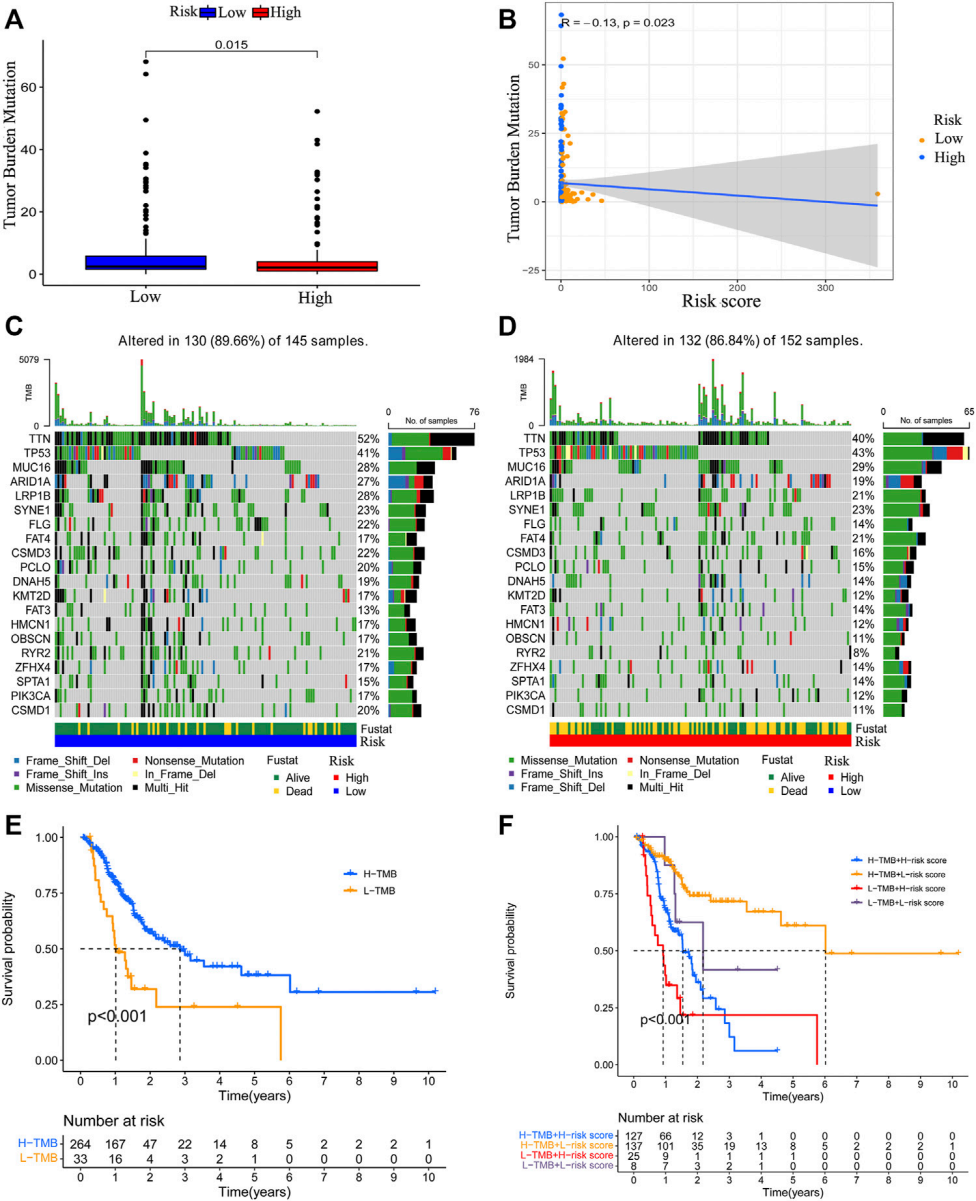
Correlation between the risk model and TMB level. The relationship between risk score value and TMB level **(A, B)**. The top 20 genes’ TMB status were visualized in two groups **(C, D)**. The survival outcome of the patients with different TMB levels **(E)**. The survival outcome of patients with low or high TMB level and lower risk score value or higher risk score value **(F)**.

To detect the relationship between the risk model and tumor mutation burden, we obtained the TMB data of GC and compared the TMB level difference between the low-risk patients and high-risk patients by using “maftools” of R. Results demonstrated that patients with a lower risk score have an elevated TMB level (Figure 9A). The risk score showed a negative correlation with TMB level (Figure 9B). The mutations status of the top 20 mutated genes in low-risk and high-risk groups were visualized, respectively. Except for TP53, MUC16, and FAT3 mutation, the mutation of other genes was higher in low-risk group patients (Figures 9C,D). Then, the survival outcomes of GC patients with different patterns of TMB and risk scores were analyzed. We observed that low-TMB patients had poorer survival outcomes (Figure 9E). In addition, low-TMB patients with higher risk scores had the worst survival outcome. On the contrary, high-TMB patients with lower risk scores had the best survival outcome (Figure 9F).

### Expression of the Pyroptosis-Related lncRNAs in GC Samples and Cells

To further find the hub genes in our risk model, we compared the expression of these 11 lncRNAs between normal tissues and tumor tissues. Interestingly, we observed that only RRN3P2, AL121772.1, AC245041.2, and AC147067.2 were differentially expressed between normal tissues and tumor tissues (Supplementary Figures S3A–K). We speculated that these four lncRNAs were hub lncRNAs associated with GC patients’ survival. Therefore, we designed the qRT-PCR primers of these four lncRNAs and further determined their expression differences between a normal human gastric epithelial cell line (GES-1) and three GC cells (AGS, MGC803, MKN45). Results demonstrated that AC245041.2 is upregulated in GC cells compared with GSE-1 cells. However, RRN3P2, AL121772.1, and AC147067.2 are downregulated in GC cells compared with GSE-1 cells (Supplementary Figures S3L–O).

## Discussion

Pyroptosis is identified as a novel type of programmed cell death which accompanies inflammatory and immune responses. Pyroptosis was reported to exert a dual role in tumor development. On the one hand, pyroptosis could suppress the proliferation and invasion abilities of various tumor cells (Masuda et al., 2006; So et al., 2018). On the other hand, pyroptosis, as a type of proinflammatory death, could lead to the release of the inflammatory mediators which might provide a proper microenvironment for tumors and thus promote tumor cell proliferation and invasion (Wei et al., 2014; Ma et al., 2016). Pyroptosis-related genes were identified as biomarkers for predicting the prognosis of tumor patients (Lin et al., 2021; Ye et al., 2021). Meanwhile, it was reported that various of lncRNAs play vital roles in numerous biological processes concerned with tumorigenesis (Peng. W.-X et al., 2017; Chi et al., 2019). In addition, a large number of lncRNAs were identified as prognostic biomarkers in multiple cancers (Jin et al., 2020; Shen et al., 2020; Wu et al., 2020). In recent years, lncRNAs were widely reported to modulate pyroptosis in many diseases including cancer (Chen et al., 2021; Huang et al., 2021; Ning et al., 2021; Wang. L.-Q et al., 2021). However, the function of pyroptosis-related lncRNAs in GC has not been explored previously.

In the present study, we first identified a novel risk model based on 11 pyroptosis-related lncRNAs (AL353804.1, AC147067.2, AP001318.2, RRN3P2, UBL7-AS1, AC018752.1, ACTA2-AS1, AL121772.1, AC005332.4, HAGLR, AC245041.2). Among the pyroptosis-related lncRNAs in the risk model, ACTA2-AS1, HAGLR, and AC245041.2 were reported to regulate the progression of various tumors, while others lncRNAs were reported for the first time. ACTA2-AS1 could function as a miR-4428 sponge to regulate the progression of colon adenocarcinoma (Pan et al., 2021). HAGLR was reported to regulate the proliferation and metastasis of lung cancer and esophageal cancer, respectively (Guo et al., 2019; Yang et al., 2019). Long noncoding RNA AC245041.2 was proved to be related to KRAS mutation and survival outcome of pancreatic cancer patients (Tian et al., 2021). In addition, HAGLR was proved to activate NLRP3 inflammasome which is associated with pyroptosis (Zhang et al., 2021). These findings indicated that the genes in our risk model were closely related to tumors. As for the correlation between 11 pyroptosis-related lncRNAs and pyroptosis, we only observed that lncRNA HAGLR could induce the activation of NLRP3 (Zhang et al., 2021), a key pyroptosis gene activated in pyroptosis, which indicated that HAGLR is associated with pyroptosis. To our knowledge, the other ten lncRNAs didn’t exhibit any obvious correlations with pyroptosis. Although the evidence of the correlation between other 10 lncRNAs and pyroptosis was not observed, our study might provide crucial clues for exploring the association between these lncRNAs and pyroptosis.

After acquiring the risk model, all patients were divided into two subgroups according to the median value of the risk score. PCA results showed that the risk model has a good efficiency in separating patients with different risk patterns. To further determine the prognostic function of the model, we analyzed survival differences between low-risk patients and high-risk patients. Results demonstrated that patients in the low-risk group have better survival outcomes than those in the high-risk group. A ROC curve was conducted to determine the accuracy of the model. We observed that the AUC values exceeded over 0.85 at one, three, and 5 years. The maximum AUC value (0.941) was detected at 5 years. Then, we further explored the function of the risk model in independent prognosis and found that risk score could be used as an independent prognostic indicator. We also conducted clinically relevant ROC curve analysis and decision curve analysis (DCA). The results showed that the risk score has elevated efficiency in clinical application than other clinical characteristics. Subsequently, we tested the prognostic function of the model in patients with diverse clinical characteristics and found that low-risk patients in all subgroups have better survival outcomes than high-risk patients. These findings suggested that our risk model could be used for the prognosis prediction of GC patients.

Pyroptosis was widely reported to exert vital functions in the tumor microenvironment, especially in the immune landscape. Many inflammatory mediators were released during pyroptosis, which might form a suitable microenvironment for the growth of tumor cells (Xia et al., 2019). Induction of pyroptosis in a small percent of tumor cells could affect the activation of T cell-mediated immune response (Wang et al., 2020). The infiltration of CD8^+^ and CD4^+^ T cells in tumors could be affected by pyroptosis (Erkes et al., 2020). These results indicated that pyroptosis might be associated with the immune landscape of tumors. Two recent studies have revealed that pyroptosis-related genes could be utilized to predict the prognosis and immune landscape of ovarian and gastric cancer (Shao et al., 2021; Ye et al., 2021). The tumor immune microenvironment exerts a crucial function in the development of cancer. Infiltration differences of various immune cells in tumors could affect the prognosis of patients (Edlund et al., 2019; Xu et al., 2020). A higher infiltration level of helper T cells and Natural kill cells is associated with better tumor prognosis (Melssen and Slingluff, 2017; Cursons et al., 2019; Kim et al., 2021). On the contrary, a higher infiltration level of macrophages, monocytes, mast cells, and neutrophils is associated with poor prognosis for patients with cancers (Cassetta and Pollard, 2018; Hu. G et al., 2018; Gao et al., 2021; Ugel et al., 2021). In addition, macrophages, monocytes, mast cells, and neutrophils were reported to promote tumor progression. Macrophages were reported to promote tumor progression, which might provide therapeutic approaches for cancers (Peng. L.-S et al., 2017; Cassetta and Pollard, 2018). Monocytes were also reported to be associated with cancer progression (Peng. L.-S et al., 2017). Mast cells could induce the release of lymphangiogenic factors and angiogenic factors and thereby promote tumor progression (Lv et al., 2019; Sammarco et al., 2019). Neutrophils are associated with tumor progression and might be used as therapeutic targets for anti-cancer therapies (Granot, 2019). These results indicated that the immune infiltration status of immune cells could affect the prognosis of patients with tumors.

To explore the relationship between the risk model and immune landscape, we analyzed the immune cells infiltration status of GC patients. We observed that there was more infiltration of NK cells and T cells in the low-risk group. However, infiltration of macrophages, monocytes, mast cells activated and neutrophils was higher in high-risk group patients. Our results indicated that high-risk patients might have poorer survival outcomes, which is consistent with the prognostic results. Therefore, our risk model has a preferable performance in predicting the immune infiltration of GC patients.

Immune checkpoints and tumor mutation burden (TMB) are another two indicators related to the immune landscape. The expression of immune checkpoint genes could affect the immunotherapy sensibility of cancer patients (Burugu et al., 2018). Patients with a higher level of TMB might be more sensitive to immunotherapy (Allgäuer et al., 2018; Zhao et al., 2019). Therefore, we analyzed the expression level of immune checkpoints and observed that the expression of CD160, CTLA4, TNFRSF14, and TNFSF15 were higher in the low-risk group. We also determined the relationship between the risk score and TMB and observed that the risk score is negatively correlated with the TMB level. The survival analyses concerned with TMB and risk score revealed that the survival outcomes of low TMB patients with a higher risk score were the worst. On the contrary, high-TMB patients with a lower risk score had the best survival outcomes. Based on these results, we speculated that low-risk patients might be more sensitive to immunotherapy. To prove our hypothesis, we acquired the immunotherapy data of GC and evaluated the differences in immunotherapy scores between the two groups. We observed that there was no difference in immunotherapy scores in CTLA4 and PD-1 double-negative patients. However, the immunotherapy score of low-risk patients with single positive CTLA4 or PD-1 and double-positive CTLA4 and PD-1 were higher, indicating these low-risk patients might be more sensitive to immunotherapy. These results further proved that our risk model is associated with the immune landscape and could be used to predict the prognosis of GC patients.

To find the hub genes in our risk model, we analyzed the expression of 11 pyroptosis-related lncRNAs between normal tissues and tumor tissues. RRN3P2, AL121772.1, AC245041.2, and AC147067.2 were revealed to be differentially expressed between normal tissues and tumor tissues. All these four genes were up-regulated in tumor tissues. Among these four lncRNAs, RRN3P2, AC245041.2, and AC147067.2 were also up-regulated in high-risk patients. To further find the key genes closely associated with GC progression, we further determined the expression of these four lncRNAs in a normal human gastric epithelial cell line (GES-1) and three GC cells (AGS, MGC803, MKN45). Interestingly, we observed that only AC245041.2 is up-regulated in GC cells compared with the GES-1 cell line. The qRT-PCR results of the other three lncRNAs were not consistent with the expression of these lncRNAs obtained from the online data. We attributed this to the following two aspects. Firstly, gastric cancer tumors have a high level of intratumoral heterogeneity (Gao. J.-P et al., 2018; Wang. R et al., 2021). The heterogeneity is mainly due to the different proportions of the stromal cells and the immune cells in tumor tissues. In addition, the heterogeneity among a variety of different tumor cells could also affect the expression levels of the gene. Secondly, the expression of lncRNAs obtained from the online data was derived based on non-paired normal tissues and GC tissues, which could also affect the expression data.

Despite that our findings revealed a key lncRNA AC245041.2 is dysregulated in GC tissues and cells, the expression of AC245041.2 in paired normal tissues and GC tissues, and the functional study on AC245041.2 are still needed, which will be explored in our future study.

## Conclusion

In conclusion, we conducted a comprehensive and systematic bioinformatics analysis and obtained a pyroptosis-related lncRNA risk model. The risk model exerted a preferable performance in predicting the prognosis of GC patients. Moreover, the risk model was associated with the immune landscape of GC patients. Our study generated a novel prognostic signature for GC patients and might offer crucial clues for future immune studies of GC.

## Materials and Methods

### Cell Culture, RNA Isolation, and Quantitative Real-Time PCR

A normal human gastric epithelial cell line (GES-1) and three GC cell lines (AGS, MGC803, and MKN45) were bought from the Type Culture Collection of the Chinese Academy of Sciences (Beijing, China). These Cell lines were cultured with RPMI 1640 (GIBCO, Carlsbad, United States) supplemented with 10% certified Fetal Bovine Serum (VivaCell, Shanghai, China).

Total RNA was isolated from GC cells and GES-1 cells by using Trizol reagent according to the manufacturer’s protocol (Takara, Japan). For the qRT-PCR assay, we synthesized cDNA using PrimerScript RT Reagent Kit (#RR037A, Takara, Japan). All mRNA primers were designed and synthesized by Sangon Biotech (Sangon Biotech, Shanghai, China). The information of primers was shown in Supplementary Table S6.

### Data Acquisition and Processing

The expression matrix and its corresponding clinical data of gastric cancer patients were acquired from the Cancer Genome Atlas (TCGA database). Patients with a survival time of more than 30 days were included. The annotation human GTF file was obtained from Ensembl (http://asia.ensembl.org) and utilized to annotate mRNA and lncRNA of the expression matrix. All methods used in our article were performed in accordance with the relevant guidelines and regulations.

### Acquiring of the Pyroptosis-Related lncRNAs

According to previous studies (Lin et al., 2021), we acquired 33 pyroptosis genes and extracted expression data of these genes from the expression matrix by using “limma” package R. Then, we identified 484 pyroptosis-related lncRNAs from annotated lncRNA expression data based on 33 pyroptosis genes by using Pearson’s correlation analysis (Pearson ratio >0.3 and *p-*value < 0.001).

### Construction of the Risk Model

After obtaining 484 pyroptosis-related lncRNAs, all patients (305 samples, the entire set) were randomly divided into two subgroups (training set:153 samples, testing set:152 samples). The training set was used for the construction of the risk model. In brief, the univariate analysis was conducted to screen prognostic pyroptosis-related lncRNAs. 34 prognostic pyroptosis-related lncRNAs were obtained. Then, the LASSO analysis and multi-cox analysis were performed to obtain the risk model based on 34 prognostic pyroptosis-related lncRNAs. Eleven pyroptosis-related lncRNAs were identified for the establishment of the risk model. The risk score value was calculated as follows:

$$Risk\;score\left(patients\right)=\sum_{k=1}^{n}coef\;value\left(gene\;k\right)*expression\left(gene\;k\right)$$

In this formula, n, k, coef value , and expression  represent the number of lncRNAs, selected lncRNA, regression coefficient value, and lncRNA expression value, respectively. The cutoff value of the risk score is 1.0076. PCA analysis was conducted for dimensionality reduction of the entire gene set, 33 pyroptosis genes, and 11 pyroptosis-related lncRNAs in the risk model based on the risk pattern of the GC patients (Xu et al., 2021).

### Validation of the Risk Model

All patients were divided into a low-risk group and a high-risk group according to the median value of risk score (1.0076). Then, Kaplan-Meier analysis was performed to explore the survival differences of patients between two groups. The accuracy of the model was determined by using a ROC curve. The survival status of the GC patients with different risk scores was further plotted. The R package of “survivalROC,” “survival,” and “survminer” was used in the above analyses. The RNA level of 11 lncRNAs in the risk model was visualized by using the “pheatmap” package of R. The effectiveness and accuracy of the model were further validated in the testing set and the entire set, respectively.

### Prognostic Function of the Risk Model

The correlation between the risk pattern and clinical characteristics of GC patients was determined by using the chi-square test. Univariate analysis and multivariate analysis were conducted to confirm the independent prognostic function of the model. A clinically relevant ROC curve and a decision curve were utilized to validate the clinical application value of the risk model. The predicted survival time of GC patients was calculated by using a nomogram. Then, the accuracy of the nomogram was determined by using a calibration curve. In the above analysis, the “rms,” “survival,” and “regplot” packages of R were used. The overall survival difference among patients with diverse clinicopathological characteristics was detected by using Kaplan-Meier survival analysis.

### Evaluation of Immune Cells Infiltration in GC Patients

An integrated TCGA immune cells infiltration data (CIBERSORT, TIMER, QUANTISEQ, XCELL, EPIC, and MCPcounter) was acquired from TIMER2.0 (https://timer.comp-genomics.org). Then, the infiltration difference of immune cells between the low-risk group patients and high-risk group patients was assessed by using the Wilcoxon test. The correlation between the immune infiltrating cells and risk score was determined by using Spearman correlation analysis. In the above analyses, the “limma,” “pheatmap,” “scales,” “ggplot2,” “ggtext,” and “ggpubr” packages of R were used.

### Prediction of Therapeutic Sensitivity in Patients With Different Risk Scores

We assessed the function of the risk score in predicting 138 chemotherapies/targeted drugs sensitivity. The pRophetic algorithm was used to evaluate the 50% inhibiting concentration (IC50) of 138 drugs. The drugs with *p* < 0.005 were exhibited. We also determined the difference in mRNA level of immune checkpoint genes between low-risk patients and high-risk patients. Then, the immunotherapy score data of the patients were downloaded from (https://tcia.at/). The therapeutic sensitivity of low-risk patients and high-risk patients to immunotherapy was assessed. The “limma,” “reshape2,” “ggplot2,” and “ggpubr” packages of R were used in the above analyses.

### Correlation Between the Risk Model and Tumor Mutation Burden

Tumor mutation burden (TMB) data was acquired from the TCGA database. Then, the correlation between TMB level and risk score value was assessed and visualized by using the “ggpubr,” “reshape2,” and “ggplot2” packages of R. The difference of TMB level between the two groups was visualized by using the “maftools” package of R. The survival difference among patients with different patterns of TMB level and risk score value was determined and visualized by using Kaplan-Meier analysis.

### Statistical Analyses

All results were acquired and generated by using R (version 4.1.0) or Perl (5.32.1.1). The statistical methods used in each part were described above.

## Data Availability

The original contributions presented in the study are included in the article/Supplementary Material, further inquiries can be directed to the corresponding authors.
