# Supplementary figures and images for: A Novel Risk Model Identified Based on Pyroptosis-Related lncRNA Predicts Overall Survival and Associates With the Immune Landscape of GC Patients
Source: Front Genet. 2022 Feb 7;13:843538. doi: 10.3389/fgene.2022.843538 (PMC8859253; doi:10.3389/fgene.2022.843538)

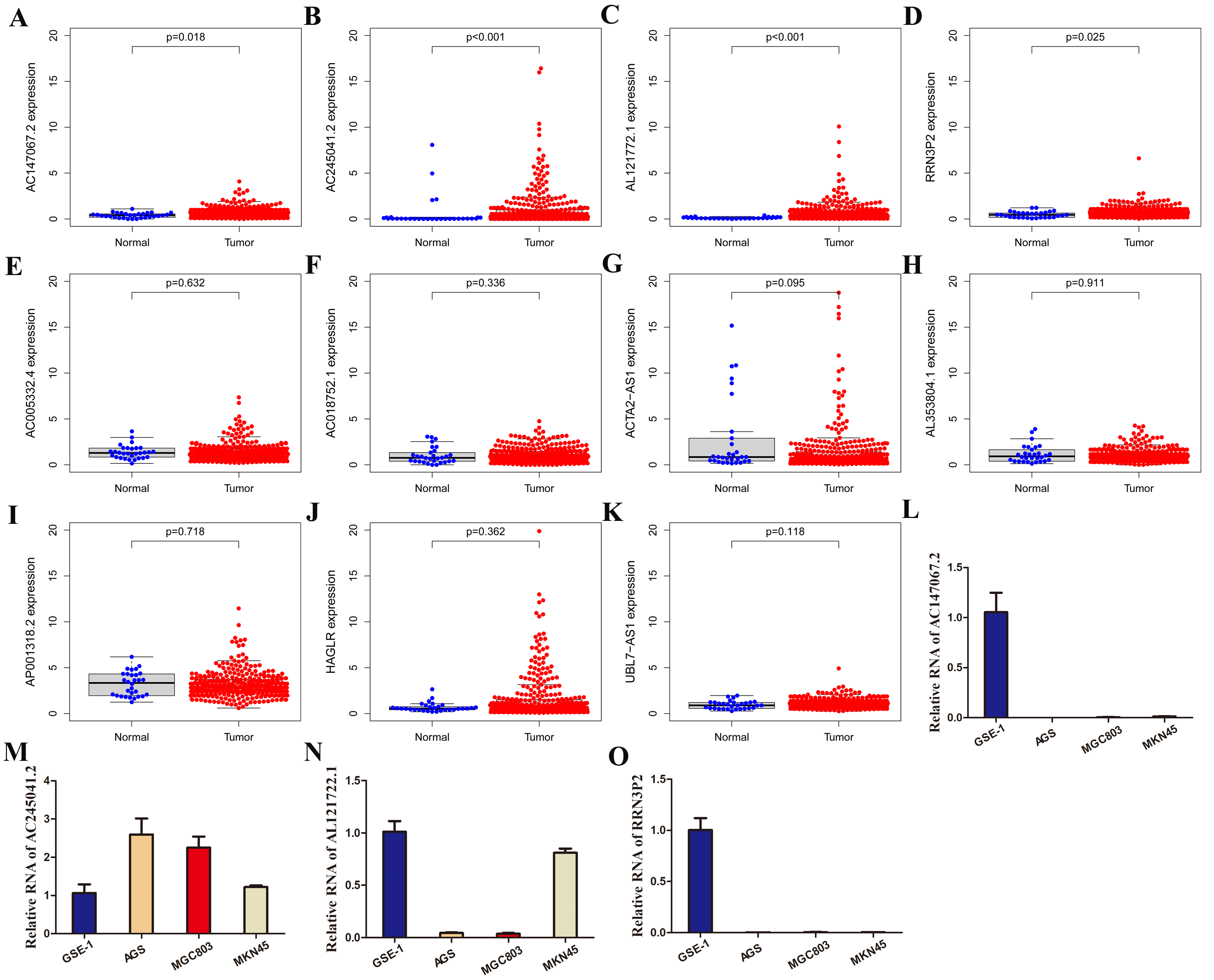

Supplement: Supplementary file 1 [file Image3.TIF]

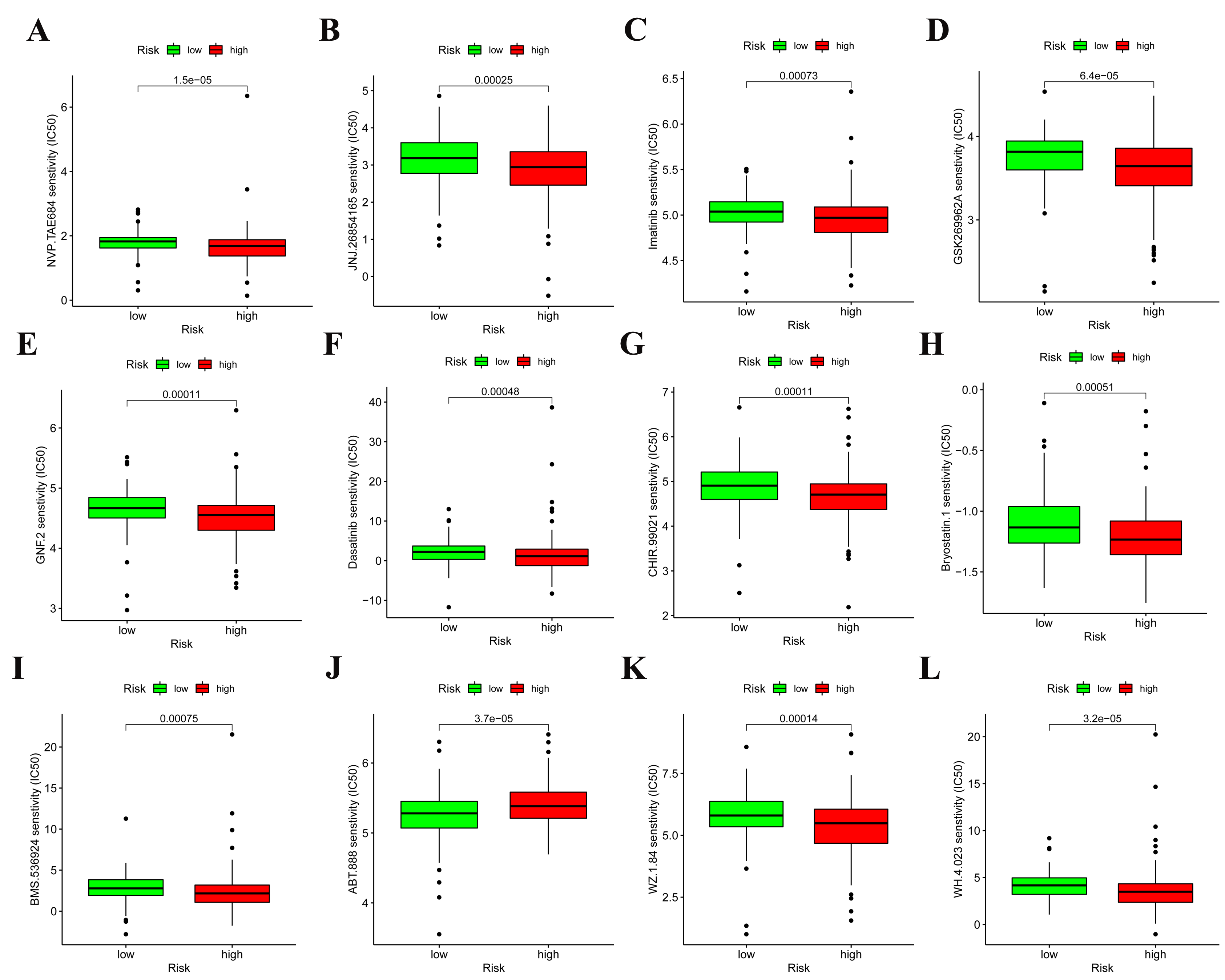

Supplement: Supplementary file 3 [file Image2.TIF]

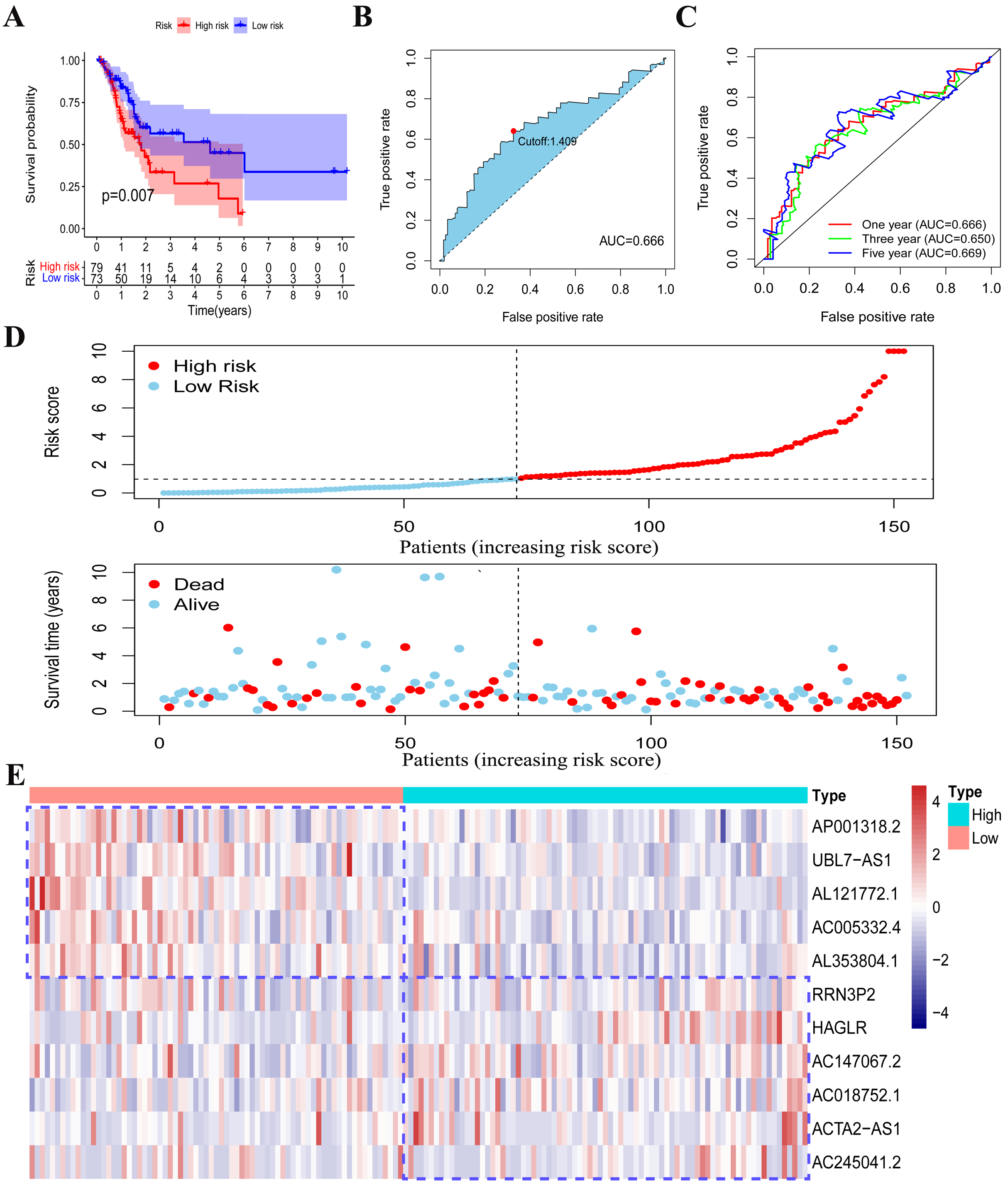

Supplement: Supplementary file 4 [file Image1.TIF]
