# Supplementary material for: A Novel Risk Model Identified Based on Pyroptosis-Related lncRNA Predicts Overall Survival and Associates With the Immune Landscape of GC Patients
Source: Front Genet. 2022 Feb 7;13:843538. doi: 10.3389/fgene.2022.843538 (PMC8859253; doi:10.3389/fgene.2022.843538)
Supplement: Supplementary file 2 [file DataSheet1.ZIP › Supplementary table 6.doc]

**Supplementary Table 6: quantitative real-time PCR primer sequences**

| Name | Primer |
| --- | --- |
| RRN3P2  AL121772.1  AC245041.2  AC147067.2  GAPDH | F: GGGGCCTTCAGGTCACTTGG  R: CGCATCTCCAGCAAACTGAGC  F: ACTGAGCTTCACATCCGCACAA  R: AAATCCACCCCATCATCGAGT  F: CATACAATGGAATGGTAGCAGAC  R: TTCAGGAACACGATTCAGAGC  F: TCCCTTATCACCGTTAGCCAT  R: CACAGTTGCACAGACGACAG  F: CTTTGGTATCGTGGAAGGACTC  R: GTAGAGGCAGGGATGATGTTCT |
